# Tunable High Spatio-Spectral Purity Undulator Radiation from a Transported Laser Plasma Accelerated Electron Beam

**DOI:** 10.1038/s41598-019-55209-4

**Published:** 2019-12-13

**Authors:** A. Ghaith, D. Oumbarek, E. Roussel, S. Corde, M. Labat, T. André, A. Loulergue, I. A. Andriyash, O. Chubar, O. Kononenko, S. Smartsev, O. Marcouillé, C. Kitégi, F. Marteau, M. Valléau, C. Thaury, J. Gautier, S. Sebban, A. Tafzi, F. Blache, F. Briquez, K. Tavakoli, A. Carcy, F. Bouvet, Y. Dietrich, G. Lambert, N. Hubert, M. El Ajjouri, F. Polack, D. Dennetière, N. Leclercq, P. Rommeluère, J.-P. Duval, M. Sebdaoui, C. Bourgoin, A. Lestrade, C. Benabderrahmane, J. Vétéran, P. Berteaud, C. De Oliveira, J. P. Goddet, C. Herbeaux, C. Szwaj, S. Bielawski, V. Malka, M.-E. Couprie

**Affiliations:** 1 Synchrotron-SOLEIL, L’Orme des Merisiers, Saint-Aubin, Gif-sur-Yvette 91192 France; 2 Université Paris-Saclay, Paris, France; 3 Univ. Lille, CNRS, UMR 8523 - PhLAM - Physique des Lasers Atomes et Molécules, 59000 Lille, France; 4 LOA, ENSTA Paris, CNRS, Ecole Polytechnique, Institut Polytechnique de Paris, 828 Bd des Maréchaux, 91762 Palaiseau Cedex, France; 5 Department of Physics of Complex Systems, Weizmann Institute of Science, Rehovot, 761001 Israel; 6 NSLS-II, Brookhaven National Laboratory, 98 Rochester St, Upton, NY 11973 USA

**Keywords:** Laser-produced plasmas, Free-electron lasers

## Abstract

Undulator based synchrotron light sources and Free Electron Lasers (FELs) are valuable modern probes of matter with high temporal and spatial resolution. Laser Plasma Accelerators (LPAs), delivering GeV electron beams in few centimeters, are good candidates for future compact light sources. However the barriers set by the large energy spread, divergence and shot-to-shot fluctuations require a specific transport line, to shape the electron beam phase space for achieving ultrashort undulator synchrotron radiation suitable for users and even for achieving FEL amplification. Proof-of-principle LPA based undulator emission, with strong electron focusing or transport, does not yet exhibit the full specific radiation properties. We report on the generation of undulator radiation with an LPA beam based manipulation in a dedicated transport line with versatile properties. After evidencing the specific spatio-spectral signature, we tune the resonant wavelength within 200–300 nm by modification of the electron beam energy and the undulator field. We achieve a wavelength stability of 2.6%. We demonstrate that we can control the spatio-spectral purity and spectral brightness by reducing the energy range inside the chicane. We have also observed the second harmonic emission of the undulator.

## Introduction

Accelerator based light sources^1^ have experienced a remarkable increase of brilliance in the X-ray domain these last decades, transforming our understanding of the world using synchrotron light. Storage ring based facilities predominantly use insertion devices, commonly known as undulators, and benefit from the improvements of the electron beam parameters in particular the energy spread and emittance. They provide high brilliance X-ray radiation that addresses the 21^*st*^ century societal challenges such as health, environment, energy, information technology and fundamental science. The radiation from electrons, wiggling in the undulator sinusoidal magnetic field, interfere constructively at the resonance wavelength and its harmonics, leading to a spectrum, consisting of sharp lines^2–11^ with a specific spatio-spectral distribution (“moon-shape” type pattern^12–15^). The generated radiation can acquire a high spectral purity and brightness, provided that the multi-electron contribution does not excessively affect on the interference process. The multi-electron degradation of the undulator spectral purity is mitigated thanks to improved electron beam performance, brought with the advent of diffraction limited storage rings^16^. Furthermore, the new laser revolution with the advent of X-Ray FEL^17^, using relativistic electrons wiggling in an undulator as a gain medium in which light is amplified due to a stimulated Compton Backscattering process^18^, brings a jump of several orders of magnitude in peak brightness. The high spectral purity and short pulses X-ray FELs open the path for deciphering un-explored ultra-fast phenomena^19^ with very high temporal resolution.

In view of miniaturizing accelerator based light sources, Laser Plasma Accelerator (LPA)^20–25^, with sub-PW class laser, serves as an attractive alternative to conventional Radio-Frequency (RF) acceleration. In an LPA, a high-power and ultra-short laser, focused into a gas target, drives a plasma wave that can trap and accelerate electrons from the ambient plasma^26–28^. LPA can deliver up to several GeV electron beams^22,29^ within a centimeter accelerating distance with low emittance^30,31^, few-femtosecond bunch length^32^ and high peak current^33–35^. While RF linacs deliver 1 nC charge beams with microradian divergence and ~0.01% energy spread, LPA still presents largely open challenges concerning the achievable energy spread^36^ at high charge operation, the initial divergence and shot-to-shot fluctuation. Indeed, the electron beam could be naturally deteriorated because of chromatic effects^37–39^. Thus, special electron beam transport, with a well-designed phase-space manipulation from source to the undulator, is required for achieving narrow undulator radiation bandwidth and making LPA based FEL^40–42^ amplification possible^43–48^. For example, the emittance growth can be mitigated either using a plasma lens or high gradient quadrupoles. In addition, a magnetic chicane can be implemented^43,45^ to reduce the slice energy spread or the use of a transverse gradient undulator^49^ to compensate the effect of the energy spread.

Up to now, the LPA-based undulator radiation^50–53^ is still limited in terms of performance: large shot-to-shot spectral and intensity fluctuations, wide relative FWHM bandwidths (7.5%^50^, 16%^52^, 22%^51^) resulting mainly from the large energy spread of the electron beam. Preliminary experimental results on undulator radiation have been observed after a dedicated manipulation transport line^48^. The full undulator features (wavelength tunability, spectral purity, stability…) suitable for taking advantage of this ultra-short undulator radiation for scientific applications have not been demonstrated so far. While the spectral purity of LPA based Compton sources^54,55^ allows for imaging applications^56,57^, the quality of the LPA based undulator radiation currently constitutes a challenge for being useable as a light source for scientific applications. As an associated prerequisite, reliable modeling of the undulator emission with a controlled electron beam is needed in view of synchrotron radiation and FEL application.

We report here that LPA electrons, in a phase-space manipulation transport line, can radiate in an undulator with properties approaching those achieved with conventional accelerators. The observed radiation presents the characteristic spatio-spectral distribution of the emitted light, exhibiting a “moon-shape” pattern as clear-cut evidence of the undulator radiation process. We then demonstrate that the electron beam control along the transport line enables us to achieve a proper reproducible wavelength tunabilty over ~100 nm range by either electron beam energy or undulator magnetic field tuning. Finally, we show that we can achieve a spectral purity of $\frac{\Delta \lambda }{\lambda }$ = 7.6% FWHM with a spectral peak brightness of 6 × 10^17^ ph/s/mm^2^/mrad^2^/0.1% BW.

## Results

### Experimental set-up and COXINEL transport manipulation line

**Figure 1:**
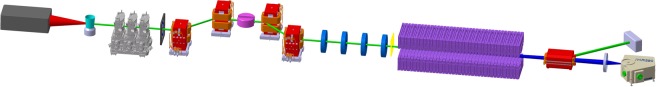
COXINEL Experimental set-up. Laser source (grey), gas jet (cyan), permanent magnet based quadrupoles (QUAPEVAs) (light grey), LANEX screen (black), electro-magnet dipoles (red) with an adjustable slit placed at the center (pink), electro-magnet quadrupoles (blue) with a 75 *μ*m-thick Aluminum foil inserted at the center to remove plasma radiation and laser beam contamination (yellow), undulator (purple), dipole magnet (red) for electron beam dump (light purple), lens (grey) focusing the undulator radiation into a UV spectrometer (light grey).

Figure 1 presents the COXINEL experimental setup. A Titanium:Sapphire laser (60 TW, 30 fs) is focused on a supersonic gas jet (99% He and 1% N_2_). The LPA is operated in the robust ionization injection regime^28^ producing electron beams with a broad energy spectrum ending at ~250 MeV, a typical charge density of ~0.5 pC/MeV and a large divergence in the mrad range. The electrons are then transported and manipulated through the COXINEL beamline^48,58^. A triplet of strong tunable permanent magnet based quadrupoles (QUAPEVAs)^59^, located immediately after the electron beam source, focuses the beam and mitigates the emittance growth. The beam is then longitudinally stretched by passing through a four-dipole magnetic chicane, where a variable width slit placed at the center selects a smaller energy range^60–62^. The optics ensures a proper energy selection via the slit (see Table 1), removal of the lateral “wings” of the transverse electron beam distribution and minimization of the beam size at the center of the undulator (see Methods). The Twiss parameters and emittance change significantly for different energies due to chromatic effects. Next, the electron beam goes into a second set of quadrupoles ensuring a focusing at the center of the undulator. The 18.16 mm period undulator can be adjusted between 4.55–30 mm gap attaining a peak field *B*_*u*_ of ~1.2 T at minimum gap^63^. The radiation is emitted in the Ultra-Violet (UV) for reference electron energies within 150–180 MeV (see Methods). Finally, the electron beam is dumped using a dipole magnet at the end of the transport line. The radiation is collected by a lens and focused at the entrance slit of a UV imaging spectrometer, enabling to map the radiation spatio-spectral distribution on a CCD camera (see Methods).

**Table 1:** Electron beam parameters at the undulator center.

E	Slit	*σ*_*γ*_	*σ*_*x*_	*σ*′_*x*_	*σ*_*z*_	*σ*′_*z*_	*σ*_*l*_	Q
MeV	mm	%	*μ*m	*μ*rad	*μ*m	*μ*rad	*μ*m	pC
176	3	3.0	800	650	240	1100	140	2.5
161	4	3.1	860	580	120	1450	135	5.6
161	3	2.6	800	570	115	1390	115	4.8
161	2	2.0	740	560	120	1290	90	3.7
161	1	1.4	680	550	130	1060	60	2.1

Electron energy spread *σ*_*γ*_, transverse sizes (in horizontal *σ*_*x*_, in vertical *σ*_*z*_), divergences (*σ*′_*x*_, *σ*′_*z*_), bunch length *σ*_*l*_ and charge Q computed from the transport considering an initial total charge of 100 pC for two different operating electron beam energies (see Methods).

### Observation of undulator spatio-spectral distribution

**Figure 2:**
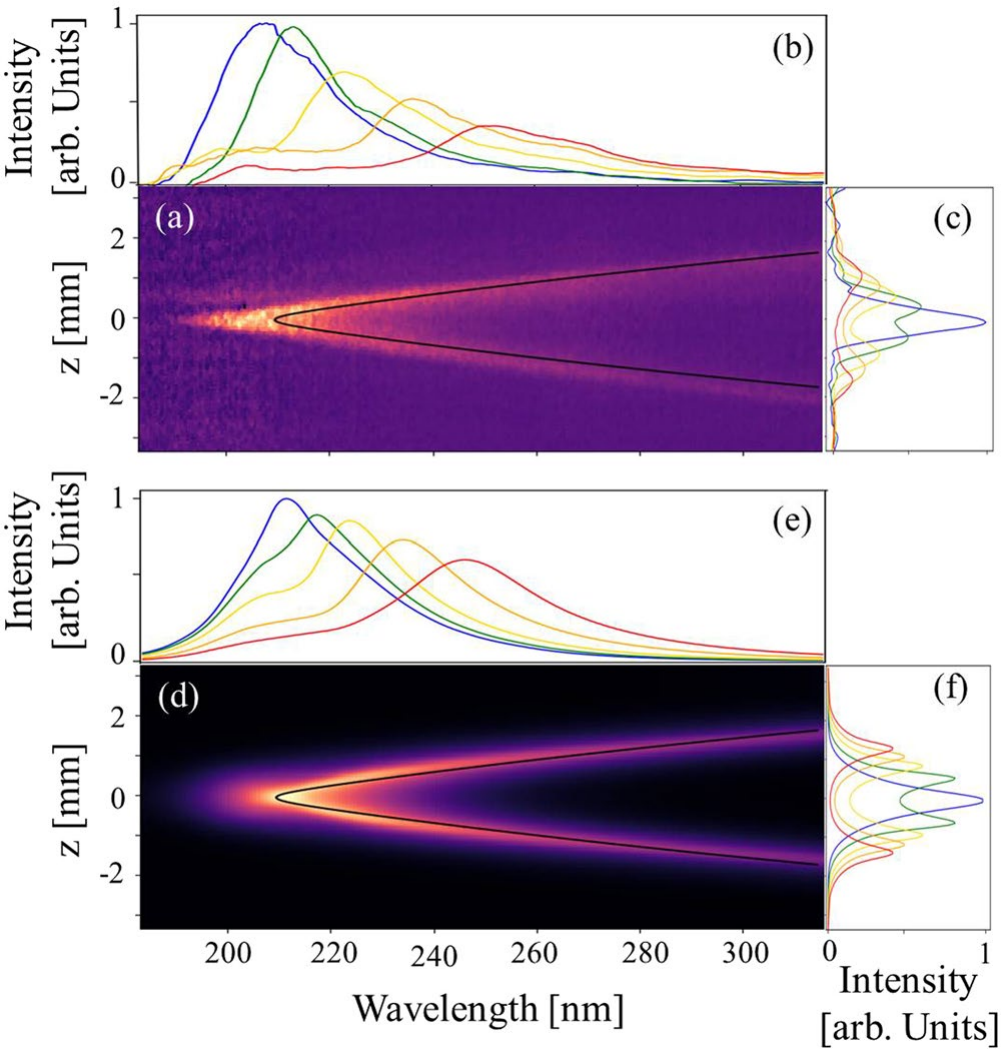
Measured and simulated undulator spatio-spectral distribution. (**a**) Single shot measurement for an electron beam energy of 176 MeV, a 5 mm undulator gap, 3 mm electron slit, 2.2 mm spectrometer slit width, and an applied calibration of the grating and CCD camera (See Methods). (**d**) Simulation using SRW with parameters of Table 1. (**b**) and (**e**) Undulator spectra for different vertical positions at *z* = 0 (blue), 0.2 mm (green), 0.4 mm (yellow), 0.6 mm (orange), 0.8 mm (red). (**c**) and (**f**) Vertical radiation profiles with cuts at different wavelengths *λ* = 208 nm (blue), 228 nm (green), 248 nm (yellow), 268 nm (orange), 288 nm (red). Black curve: fit of the undulator resonance wavelength taking into account the chromatic aberrations of the lens (See Methods).

Figure 2(a) shows a single shot measurement of the spatio-spectral distribution of the UV light for an undulator gap of 5 mm. The undulator radiation is emitted at a resonant wavelength *λ* = *λ*_*u*_(1 + *K*_*u*_^2^/2 + *γ*^2^*θ*^2^)/2*nγ*^2^, where *λ*_*u*_ is the undulator period, *K*_*u*_ the deflection parameter (*K*_*u*_ = 93.4 *B*_*u*_[T] *λ*_*u*_[m]), *γ* the Lorentz factor, *θ* the observation angle and n the harmonic number. The image exhibits a typical undulator “moon-shape” pattern resulting from the off-axis emission^12–14^. The off-axis radiation at vertical position (|*z*| > 0) is red-shifted due to the *γ*^2^*θ*_*z*_^2^ term in the resonance wavelength relationship (for example 232 nm for |*z*| = 0.6 mm compared to 210 nm on-axis) and has lower intensity. The effect is more pronounced for larger angles of observation. The measurement is compared to simulation (see Fig. 2(d)) performed with the SRW code^6^. The beam parameters at undulator entrance are computed using the measured initial electron beam parameters transported along the beamline (see Methods). The far-field undulator radiation is computed separately for each energy slice and the resulting intensities are summed. The lens imaging ratio is applied including its chromatic corrections. The simulation and measurement show similar “moon-shape” patterns. The triangular shape, slightly deviating from the usual parabolic behaviour due to the *γ*^2^*θ*_*z*_^2^, results from the chromatic effects of the lens (see Methods).

Figure 2(b,e) show the vertical angular dependance of the spectra with the image cuts at various *z*. The on-axis (*z* = 0) spectrum is peaked at the resonance wavelength of 208 nm with a 13.1% relative FWHM bandwidth, larger than the natural homogeneous linewidth ((Δ*λ*/*λ*)_*hom*_ = 1/nN with N the number of undulator periods (0.84% for N = 107)) by one order of magnitude. This so-called inhomogeneous broadening results from the multi-electron contribution, and thus from the electron beam parameters. The relative energy spread *σ*_*γ*_ symmetrically widens the spectral line, as (Δ*σ*/*γ*)_*σγ*_ ≃ 2*σ*_*γ*_ (~14% FWHM for *σ*_*γ*_ of 3% RMS). The emittance broadens the spectral flux distribution integrated over z mainly on the “red” side. At z = 0, only the horizontal divergence ${(\frac{\Delta \lambda }{\lambda })}_{{\sigma ^{\prime} }_{x}}\simeq \frac{{\gamma }^{2}{\sigma }_{x}^{^{\prime} 2}}{1+{K}_{u}^{2}/2}$ (~8% for 0.65 mrad RMS) brings a non-negligible contribution. The vertical radiation profiles for increasing wavelengths, shown in Fig. 2(c,f), exhibit first a Gaussian type distribution (*λ* = 208 nm) followed by a hole dip in the center (*λ* = 228 nm) and a donut shape (*λ* = 268 nm and above), as typically observed for undulator radiation^9,15^.

### Undulator radiation tunability

**Figure 3:**
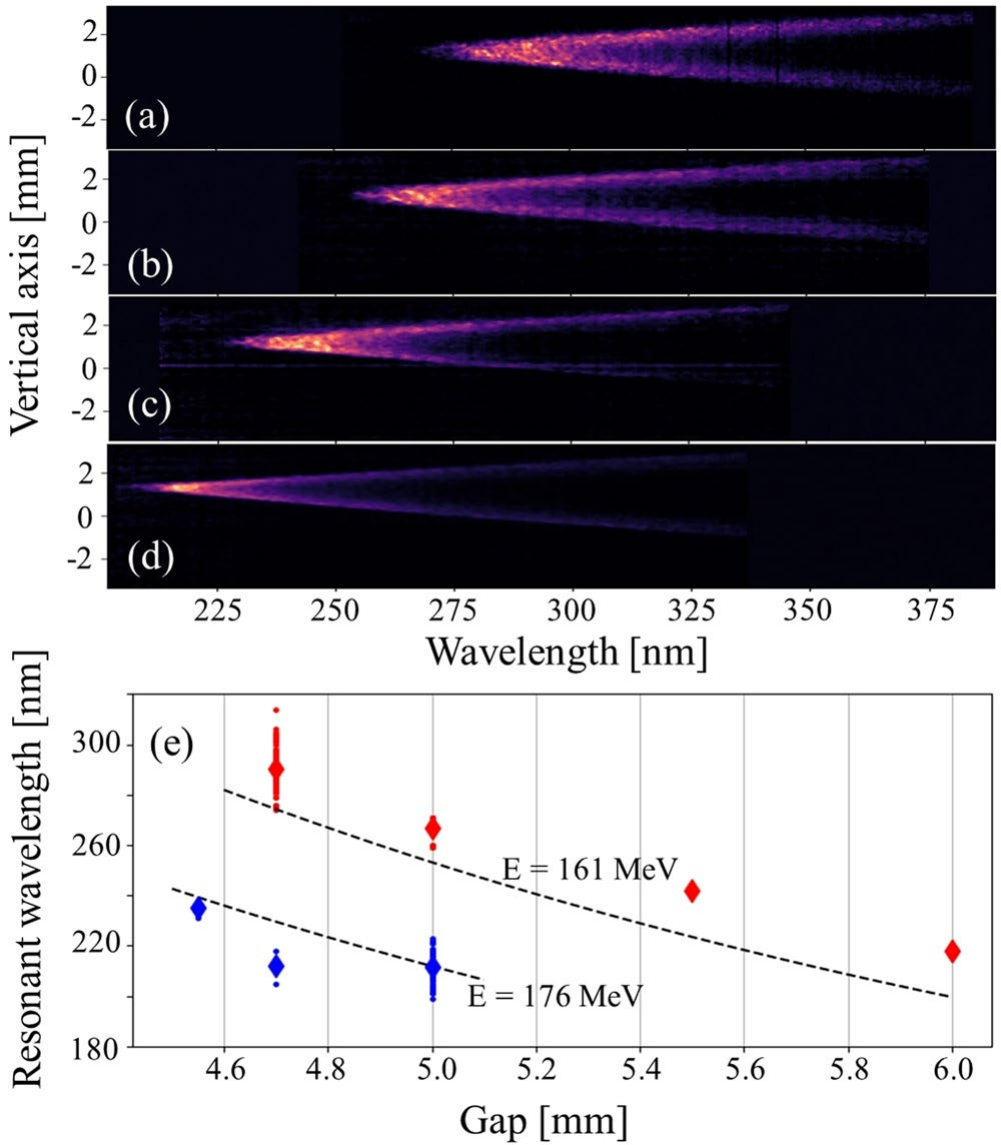
Wavelength tunability by undulator gap and energy change. Single shot spatio-spectra distribution measured for 161 MeV beam energy at different gaps: (**a**) 4.7 mm, (**b**) 5 mm, (**c**) 5.5 mm, (**d**) 6 mm, with an electron slit of 1 mm and a spectrometer slit of 2.2 mm. (**e**) Measured and theoretical (dashed) resonant wavelength versus undulator gap: 161 MeV (red) and 176 MeV (blue).

The undulator radiation tunability, one of the major undulator properties, is then explored. The radiated wavelength is independently varied either by changing the undulator gap, or through focusing a different energy by quadrupole gradients and chicane strength adjustments^48^. Such an undulator wavelength control corresponds to what is currently achieved with conventional accelerators. Figure 3(a–d) show the measured spatio-spectral distribution for different undulator gaps. The smaller the gap, the larger the resonant wavelength. Figure 3(e) displays the evolution of the measured resonant wavelength versus gap for two different energy settings. The behaviour shows good agreement with theoretical curves calculated using the measured magnetic field gap dependence (see Methods). The discrepancy between theory and experiment in the 161 MeV case could be a result of the laser degradation (orbit change leading to a variation in electron beam energy and misalignment in the undulator); a vertical misalignment of the electron beam with respect to the undulator axis (a 0.5 mm deviation leads to 1.5% field variation); the uncertainty of the undulator peak field at 4.7 mm gap extrapolation (see Methods) (a 5% field deviation corresponds to a ~7% resonant wavelength variation). The tunability is typically achieved between 210 nm and 300 nm. The photon flux increases for smaller gap, but the spectral brightness is maximum at a gap of 4.5 mm (K = 2).

### Undulator radiation wavelength stability

**Figure 4:**
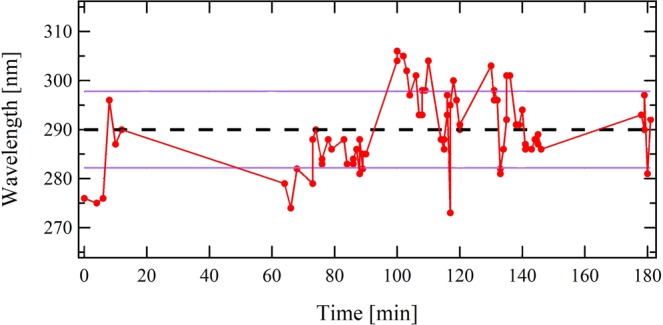
Resonant wavelength stability. Undulator resonant wavelength measured during 60 successive shots over 3 hours for an undulator gap of 4.7 mm and different electron slit widths. Average value (dashed), standard deviation (purple).

The stability of the undulator resonant wavelength was investigated. The electron beam manipulation transport line controls the proper focusing of the electron beam energy of interest in the undulator, and the slit selects a given electron beam energy range. Figure 4 displays the undulator resonant wavelength evolution during 3 hours. The wavelength is centered, on average, at 290 nm, with a RMS value of 7.8 nm over 60 shots, corresponding to a 2.6% stability.

### Undulator radiation bandwidth control

**Figure 5:**
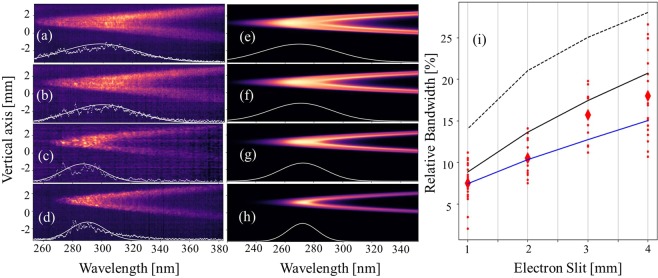
Undulator spatio-spectral distribution dependance on the electron beam energy selection. Single shot measured spatio-spectral distributions for a 4.7 mm undulator gap (with caibration) while varying the electron slit width: 4 (**a**), 3 (**b**), 2 (**c**) and 1 mm (**d**) with a 2.2 mm spectrometer entrance slit. Simulated spectra using SRW for a magnetic field of 1.17 T, with beam parameters taken from the simulations of the corresponding electron beam distribution transported along the line (see Table 1 for 161 MeV) for slit widths of 4 (**e**), 3 (**f**), 2 (**g**) and 1 mm (**h**) with their corresponding on-axis spectra (white curves). (**i**) Measured (red), analytically estimate of energy spread contribution (blue), analytically estimate of all the contributions (dashed) and simulated (line) FWHM relative bandwidth of the on-axis spectra.

After having evidenced the typical features of the measured undulator radiation, the control of the spectral bandwidth is then examined. Figure 5(a–d) show the undulator spatio-spectral patterns, with the corresponding appended on-axis spectra, measured while shaping the beam parameters. As the slit is closed in the magnetic chicane, the beam energy spread, size and divergence in the undulator are reduced (see Table 1) and, accordingly, the corresponding measured moon-shape thickness decreases. Without the slit, the energy range is broad, the resonant wavelength spans a large range and the moon-shape patterns from different energy electrons are overlapped, resulting in a strong smearing of the spatio-spectral distribution. Figure 5(i) shows the measured on-axis radiation bandwidth versus chicane slit widths. For the 1 mm slit width, the average relative bandwidth is found to be 7.6% with a standard deviation of 2% over 21 shots and the lowest undulator spectral bandwidth achieved is 2%.

These measurements can be first interpreted with an analytic approach as plotted in Fig. 5(i) using the average parameters in Table 1 with two extreme cases. In the first limiting case (blue line), where only the inhomogeneous broadening of the energy spread and horizontal divergence is considered, the observed trend of the linewidth increase is well reproduced, while the analytic estimate stands below the measured data for larger slit width since some of the contributions are neglected. The second limiting case (black line), where all contributions (emittance and energy spread) to the inhomogeneous broadening are taken into account (quadratic sum), shows a homothetic evolution same to the experimental one: the slope is mainly determined by the energy spread, with an additional linewidth widening due to the beam size and divergence increase for larger slit width. This second limiting case is situated above the measurements, since the assumption of considering separately the emittance and energy spread contributions becomes less valid for large energy spread values. The two extreme analytic cases surround the measurements. A more precise analysis of the measurements can be performed using a comparison with the simulations from SRW with the electron beam distribution at the undulator center for different electron slit widths (see Methods), as shown in Fig. 5(e–h). A quantitative agreement is achieved between measurements and simulations as shown in Fig. 5(i). The simulated bandwidths (black solid line) produce an improved agreement to the measured ones (compared to the analytic estimates), due to a proper multi-electron treatment.

The undulator spatio-spectral pattern has been successfully controlled via selecting a specific energy range of the electron beam in the transport line while transmitting the charge of the electrons at the energy of interest, and cleaning the ones for lower and higher energies. As a result, the spectral purity of the undulator radiation has been improved. The coherence length of the radiation is increased by a factor of 2.3 from the 4 mm electron slit case (4.7 *μ*m) to the 1 mm (10.8 *μ*m) one. Controlling directly the spectral bandwidth with the electron beam energy spread, instead of using a photon monochromator, enables us to preserve the radiation wavefront and avoid intensity reduction.

### Undulator spectral brightness

The behavior of the photon angular flux and spectral brightness, i.e. the photon flux over transverse and longitudinal phase space area, is then analyzed versus the electron energy control. The calculations (see Methods) are done using the LPA average beam parameters deduced from the transport simulations of Table 1 (see Methods). The peak spectral brightness is calculated to be ~2 × 10^17^ ph/s/mm^2^/mrad^2^/0.1% BW and ~6 × 10^17^ ph/s/mm^2^/mrad^2^/0.1% BW using average bandwidths for an electron slit width of 4 mm and 1 mm, respectively. Indeed, for smaller electron slit widths, the total beam charge is reduced, whereas the charge for the energy of interest remains practically the same. Spectral bandwidth is narrowed due to energy spread and emittance terms, where chromatic growth is mitigated by the energy control. In consequence, the peak photon beam brightness increases. Taking the 10^*th*^ percentile of the bandwidth measured in the 1 mm slit case (3.4%), the maximum calculated spectral brightness is found to be ~1 × 10^18^ ph/s/mm^2^/mrad^2^/0.1% BW.

### Observation of second harmonic

**Figure 6:**
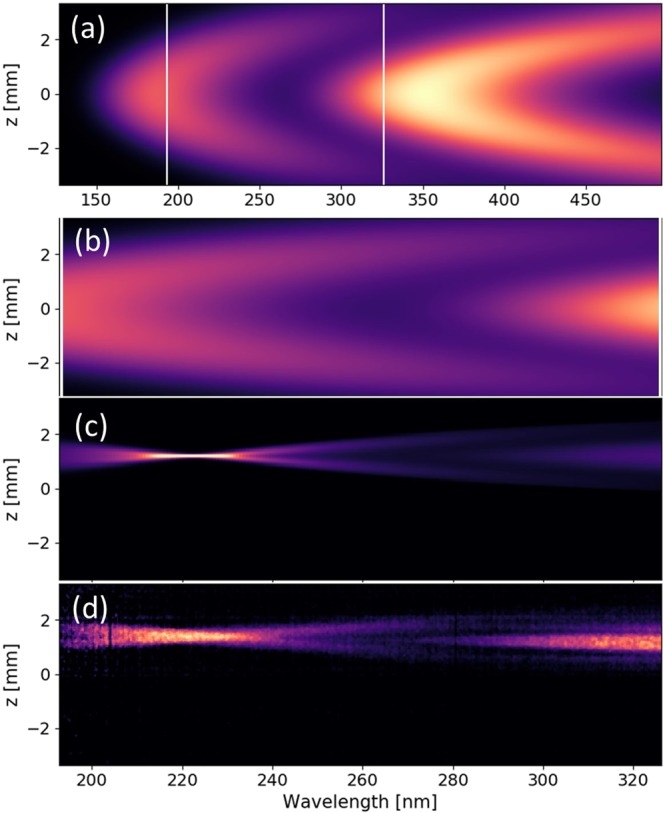
Observation of the second undulator harmonic. Undulator radiation spatio-spectral distribution at a gap of 4.7 mm, electron slit opened at 4 mm and spectrometer slit at 2.2 mm. (**a**) Simulation showing the first and second harmonic, (**b**) zoom of (**a**), (**c**) simulation including chromatic effects of the lens on (**b**), (**d**) calibrated measurement.

One of the characteristic features of planar undulators is the high intensity emission on the harmonics, provided the deflection parameter is not too small. Considering the limited spectral range of the used spectrometer, the emission on the second harmonic is explored. Figure 6(a,b) present a simulation using the average beam parameters for the 4 mm slit case (see Table 1). The second harmonic is visible on-axis (175 nm). The single-electron undulator radiation at even harmonics is known to be suppressed in the direction along the electron motion axis, because the emitted electric field at these harmonics is anti-symmetric with respect to transverse position/angle in the plane containing the electron trajectory^64^ and a significant photon flux is emitted off-axis at even harmonics. The observed on-axis radiation at 175 nm results from the contribution of the finite electron beam emittance (angular divergence at the observation in the far field and transverse size at the observation in a plane of source imaging). Figure 6(d) shows a measurement of undulator radiation, where the moon shape of the second harmonic is twisted (closed onto itself) due to chromatic effects of the lens, forming a “ribbon” type pattern. The maximum intensity is observed at ~225 nm, where the focal length of this particular wavelength is equal to the distance between the lens and the spectrometer slit (see Methods). Figure 6(c) displays the simulated undulator radiation of (b) after introducing the chromatic effects. A good agreement is found with the measurement.

## Discussion

We have examined the characteristics of LPA generated undulator radiation after a manipulation beam line. We have shown that it can exhibit the distinguished properties currently observed on conventional accelerator light sources, with the particular spatio-spectral dependance leading to a moon-shape pattern. The proper handling of the energy of interest along the COXINEL line ensures a stability of the resonant wavelength within 2.4%. A ~100 nm tunability of LPA based undulator radiation has been achieved by varying the undulator gap and electron beam energy. Furthermore, the control of the undulator radiation has been accomplished via chicane and slit combination, resulting in a small radiation bandwidth down to 7.6% FWHM and achieving a high peak photon beam spectral brightness up to 6 × 10^17^ ph/s/mm^2^/mrad^2^/0.1% BW. The satisfactory comparison between measurements and simulations using electron beam longitudinal and transverse distributions make us confident in future predictions of undulator radiation. The achieved agreement demonstrates a new capability in handling the LPA electron beam and transporting it to the undulator for a high photon beam brilliance.

Although the laser plasma acceleration set in a robust configuration (ionization injection) usually delivers electron beams with large energy spread and divergence typically above 1 mrad RMS, the control of the undulator spontaneous emission after a dedicated transport manipulation line constitutes an acquired maturity and a major step forward. One can extrapolate these results to a 1 GeV beam with initial typical parameters achieved^21,22,29,34,35^ (*σ*′_*z*_ = 2 mrad, *σ*_*γ*_ = 2.5%) with 50 pC charge, and re-optimizing the COXINEL line to transport these high energy beams with new magnetic element settings. For a 1 mm electron slit, the peak spectral brightness from the present undulator could reach ~4 × 10^21^ ph/s/mm^2^/mrad^2^/0.1% BW at a resonant wavelength of 6.5 nm. If one would replace the undulator by a LUNEX5 type (3 m long cryogenic undulator of period 15 mm and *K*_*u*_ ~ 2^65^), the peak brilliance is calculated to be ~1 × 10^22^ ph/s/mm^2^/mrad^2^/0.1% BW at a resonant wavelength of 7 nm, as compared to those achieved using high harmonic in gas (10^19^–10^23^ ph/s/mm^2^/mrad^2^/0.1% BW)^66^ and synchrotron radiation (10^19^–10^25^ ph/s/mm^2^/mrad^2^/0.1% BW)^67^.

These results pave the way for providing reliable ultra-short LPA based X-ray undulator radiation for serving the scientific user community in a configuration designed for short wavelength operation, as planned on ELI (Extreme Light Infrastructure)^68^.

## Methods

### Laser plasma accelerator

The laser-wakefield accelerator is driven by a Titanium:Sapphire laser system at Laboratoire d’Optique Appliquée, which delivers 30 fs (FWHM) pulses of 1.5 J energy at a central wavelength of 800 nm with a repetition rate of 1 Hz. The laser beam is focused by an off-axis parabola into a gas mixture composed of 99% He and 1% N_2_. A retractable electron spectrometer, placed after the supersonic gas jet with 3 mm exit diameter, measures the electron beam divergence along the vertical axis (perpendicular to the laser polarisation axis), initial energy spectrum and the charge density. A first electron beam imager located 64 cm away from the electron source enables to measure the beam horizontal and vertical divergences as well as electron beam pointing stability.

**Figure 7:**
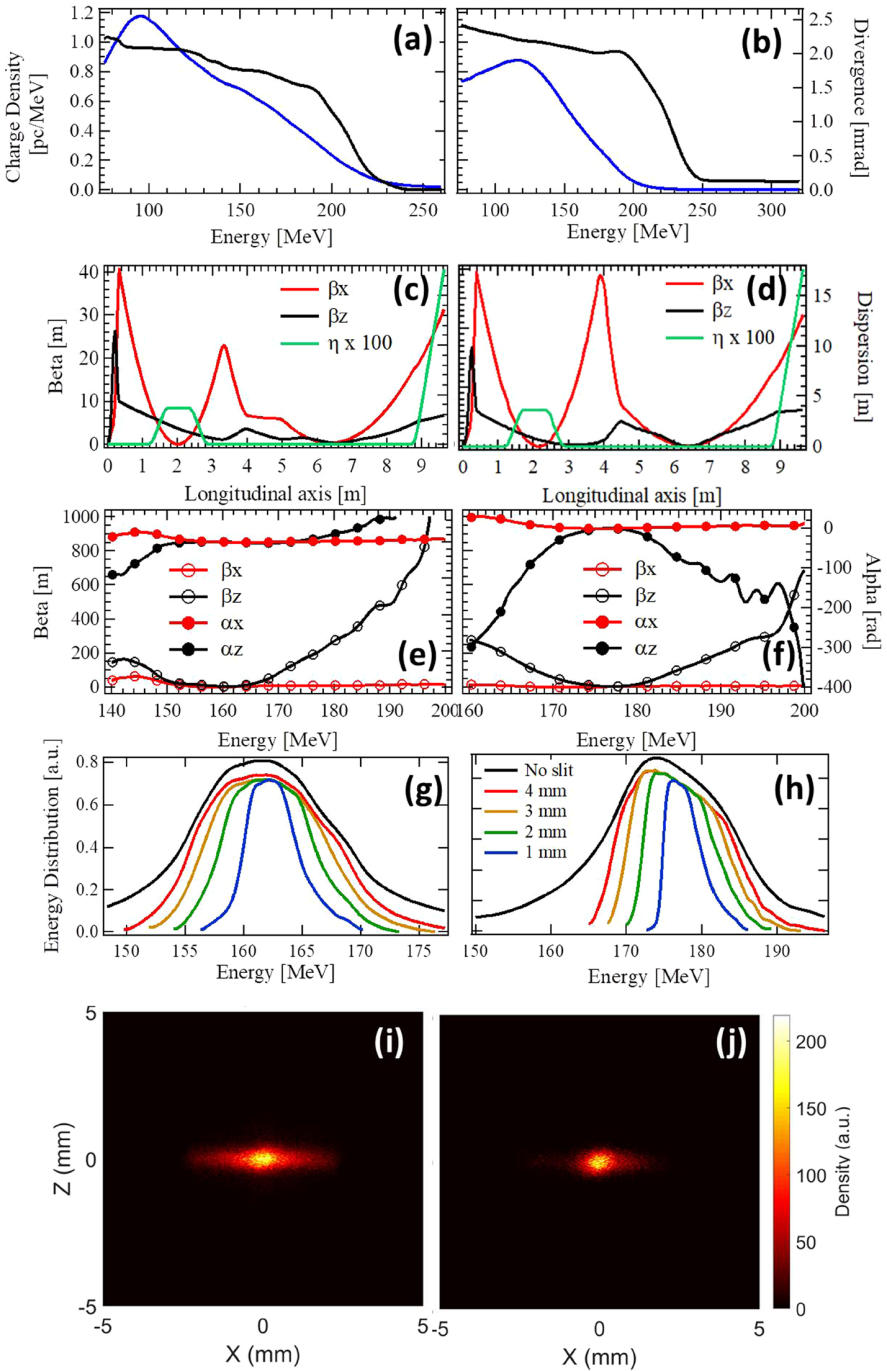
Measured electron beam charge distribution (blue), vertical divergence (black) for 161 (**a**) and 176 (**b**) MeV, with *σ*′_*x*_/*σ*′_*z*_ = 1.56. Twiss parameters (betatron and dispersion) evolution simulation for a monoenergetic electron beam of 161 (**c**) and 176 (**d**) MeV along the transport line. Twiss parameters (beta and alpha) at the undulator center versus energy slice for the 161 (**e**) and 176 (**f**) MeV cases. Energy distribution at the undulator center for different slit widths: no slit (red), 3.2 (orange), 2.2 (green), 1 (blue) mm width for the 161 (**g**) and 176 (**h**) MeV cases. Electron beam transverse distribution at undulator center for the 161 MeV: (**i**) no slit and (**j**) 1 mm slit. Parameters for the transport calculations: 1 mm.mrad initial emittance, 1 *μ*m longitudinal size, 10^6^ macroparticles, 4.3 mm chicane strength. In the 176 (resp. 161) MeV case, QUAPEVA 1 of 40.7 mm magnetic length: +104.1 T m^−1^ (resp. 113.5), QUAPEVA 2 of 44.7 mm magnetic length: −103.1 T m^−1^ (resp. −111.3), QUAPEVA 3 of 26 mm magnetic length: +96.4 T m^−1^ (resp. 103.4). QUAPEVA skew contribution (ratio of skew gradient over normal gradient) of +1.5 × 10^−3^ (QUAPEVA 1), −0.3 × 10^−3^(QUAPEVA 2), −0.7 × 10^−3^ (QUAPEVA 3) with a field variation of 2% at 4-mm radius due to a dodecapole component for the three QUAPEVA. Electromagnetic quadrupole gradients at 176 (reps. 161) MeV: −0.01, 4.7, −4.4, +0.29 T m^−1^ (4.15, −3.45, −0.13,+ 1.7 T m^−1^) for QEM 1, 2, 3, 4.

Two experimental settings have been used for the photon measurements, with a beam transport at 161 MeV (see Fig. 7(a)) and at 176 MeV energy (see Fig. 7(b)). In the 176 MeV case, the average vertical divergence measured on the spectrometer presents an average of 3.2 mrad RMS (Standard Deviation (SD) of 0.5 mrad) over 20 shots over the whole distribution, and drops to 2.1 mrad RMS (SD of 0.3 mrad over 20 shots) for the 176 ± 5 MeV slice, with an average charge of 3.1 ± 1 pC within this slice. The electron beam pointing stability, measured on the first screen, is 1.2 mrad over 20 shots. In the 161 MeV case, the average vertical divergence measured on the spectrometer presents an average of 1.95 mrad RMS (SD of 0.3 mrad) over 40 shots over the whole distribution, and of 1.85 mrad RMS (SD of 0.2 mrad over 40 shots) for the ±5 MeV slice, with an average charge of 6.0 ± 1 pC within this slice. The electron beam pointing stability, measured on the first screen, is 1.2 mrad over 5 shots.

### The electron beam transport in the COXINEL line

The COXINEL line uses permanent magnet based quadrupoles with tunable high gradient (QUAPEVAs)^59,69–71^, providing a tunability of ~45% within 10 *μ*m magnetic center change. The relative gradient precision of 6 × 10^−4^ results from the stretched wire^72^ measurements.The QUAPEVAs are mounted on translation stages, that are used for the beam pointing alignment procedure^48^. The magnetic chicane consists of four ×25 mm yoke gap water-cooled dipoles creating a 0.55 T magnetic field, measured with relative error of 1.8% for 150 A. Inside the chicane is inserted a removable slit of variable width *l* (up to 4 mm) at a horizontal position of 32 ± 0.1 mm corresponding to the 176 MeV energy. The slit, machined in a stainless steel 304L cylinder of 35 mm diameter and mounted on an under-vacuum plunger can be rotated with a MagicDrive (MD40SSES1X000Z) coupled with a stepper motor. The slit width calibration is given by: *l*[mm] = −0.5256 × (*θ* + *θ*_*offset*_) + 4.0034, with *θ* the rotation angle and *θ*_*offset*_ the offset angle, using the electron beam transmission along the line for the *θ*_*offset*_ estimation (−1.6° for 176 MeV and 0° for 161 MeV) and the CATIA model for the slope evaluation. Two Turbo Integrating Current Transformers (T-ICT from Bergoz, with 10 fC noise) after the electron generation chamber and at the exit of the undulator, measure the electron charge, giving consistant measurements with the charge density measured on the electron spectrometer.

Electron beam transport is tuned with BETA code^73^ up to the second order, with multi-particle tracking code for high order non-linear effects and collective effects such as Coherent Synchrotron Radiation^74^. Hard edge models are used for the magnets and apertures of the vacuum chamber along the line are included. For the simulations, the electron beam distribution (charge, divergence) is deduced from an average of the measured ones prior to and after the data measurements. The optics, plotted in Fig. 7(c,d), selects a smaller range of energies and minimizes the beam size at the center of the undulator thanks to the slit. The Twiss parameters at the undulator center are given in Fig. 7(e,f). The energy spread is reduced for smaller slit width (see Fig. 7(g,h) and Table 1), whereas the lateral wings of the transverse electron beam distribution are removed (see Fig. 7(i,j)).

The initial electron beam distribution is not measured simultaneously with the undulator pattern since it would intercept the beam, so it becomes a source of error in the modeling since the distribution can vary from shot-to-shot. The sensitivity to the parameters along the electron beam transport and the undulator radiation emission is considered. Simulations with emittance of 0.2 *π* mm . mrad lead to a 4% change of the radiation spectral bandwidth in the 4 mm electron slit case. A 0.5 mrad RMS divergence difference in the initial distribution results in 10% (15%, 7%) change in energy spread (respectively for transverse beam sizes and divergences), resulting in a change of 1.3% of the relative bandwidth for the 4 mm electron slit. These assumptions do not significantly affect the result and are reduced for smaller electron slit width.

### The undulator

The photon source is a 2 m long hybrid cryo-ready undulator of 107 periods (18.16 mm) operating at room temperature with adjustable gap^63^ built at Synchrotron SOLEIL. It consists of *Pr*_2_*Fe*_14_*B* magnets (remanence field of 1.32 T and coercivity of 1930 kA/m at room temperature) and Vanadium Permendur poles (field saturation at 2.35 T). The magnetic field is measured using a Hall probe with a precision of 0.5 Gauss and computed using RADIA^75^. At minimum gap *g* of 5 mm, the corresponding peak field, *B*_*peak*_, is 1.1 T. In the experiment, the undulator was closed to a gap of 4.55 mm due to the weak spectrometer response at wavelengths below 200 nm. Thus, the peak field at gaps smaller than 5 mm have been estimated by extrapolating from the measured field. *B*_*peak*_ versus *g* measured is fitted with ${B}_{peak}=a.\exp [b\frac{g}{{\lambda }_{u}}+c{(\frac{g}{{\lambda }_{u}})}^{2}]$ for gaps between 5 mm and 10 mm, where (a, b, c) = (3.37, −4.34, 1.12).

### Lens

The lens used (eSource Optics CF5025LCX) is of spherical shape made of Calcium Flouride (CaF_2_) and placed at a distance of 2.5 m from the undulator exit. The focal length *f* depends on its refractive index *n* as $\frac{1}{f(\lambda )}=\frac{n(\lambda )-1}{R}$ with *R* = 108.5 mm the radius of curvature of the lens. The chromatic dispersion is developed using the Sellmeier coefficients as $n{(\lambda )}^{2}-1=\frac{{B}_{1}{\lambda }^{2}}{{\lambda }^{2}-{C}_{1}^{2}}+\frac{{B}_{2}{\lambda }^{2}}{{\lambda }^{2}-{C}_{2}^{2}}+\frac{{B}_{3}{\lambda }^{2}}{{\lambda }^{2}-{C}_{3}^{2}}$ and *B*_1,2,3_, *C*_1,2,3_ with values from^76^. *B*_1_ = 0.5675888, *B*_2_ = 0.4710914, *B*_3_ = 3.8484723, *C*_1_ = 0.050263605 *μ*m, *C*_2_ = 0.1003909 *μ*m, *C*_3_ = 34.649040 *μ*m

### Photon spectrometer

The undulator radiation is focused with the lens onto the entrance slit of the spectrometer (Horiba IHR320) located at distance *d* from the slit, that can be varied from 80 *μ*m up to 2.2 mm. The spectrometer, equipped with a 600 groove/mm grating with an average reflectivity of ~45% (200–300 nm) and a linear dispersion of 4.917 nm/mm, enables to map the radiation vertical spatio-spectral distribution with a magnification factor from entrance slit to CCD camera *G* = 1.1 on a UV-sensitive CCD (Horiba instruments - SYNAPSE - 354308) consisting of 1024 × 256 pixels with a size of 26 *μ*m covering an area of 26.6 mm × 6.7 mm (133 nm × 6.7 mm) and a quantum efficiency of ~60% for wavelengths between 200 nm and 300 nm. The resolution variation versus slit width, measured with two lasers (green and red) is fitted with *cs*_*w*_ + *d*, where *s*_*w*_ is the slit width in mm, *c* = 5.34 nm/mm and *d* = 0.031 nm. For the data analysis, the background noise was removed, a median filter applied and convoluted with the spectrometer response.

### Modeling of the undulator radiation

The electron beam parameters deduced from the measured distribution and transported along the line are used for the undulator radiation modeling using SRW code^6^ in the far-field region. A so-called slicing method is used, where radiation of each electron energy slice is computed separately with its corresponding parameters (divergence and size), and then all the spectra are added up taking into account the slice energy distributions. Ray optics is then applied to the computed undulator radiation, assuming that the lens position is in the far-field. The undulator radiation emitted with an angle *θ*_*z*0_ impinges the lens at a height *z*_1_ = *D*_lens_*θ*_*z*0_ + *h*, with *h* the offset between the undulator radiation optical axis and *D*_lens_ the distance between the undulator center and the lens. Using the matrix formalism and the chromatic aberration effect, the wavelength-dependent conversion from *θ*_*z*0_ to spectrometer CCD vertical *z*_*CCD*_:

1 $${z}_{CCD}=G\times [{z}_{1}(1-d/f(\lambda ))+d{\theta }_{{z}_{0}}]+H,$$

with *H* the offset between the camera center and the lens optical axis. The measured moon-shape pattern of Fig. 2(a) is fitted by using the resonant wavelength relationship and Eq. 1 to deduce d, h, H and the electron beam energy.

### Spectral brightness

The spectral peak brightness is estimated using expression given in^77^, with analytical calculation of the flux (benchmarked with SRW code), the phase space transverse dimension, peak current deduced from Table 1 and measured undulator relative bandwidth.

## Data Availability

The data that support the findings of this study are available from the corresponding authors upon reasonable request.
